# Porcine Reproductive and Respiratory Syndrome Virus Prevalence and Pathogenicity of One NADC34-like Virus Isolate Circulating in China

**DOI:** 10.3390/microorganisms13040796

**Published:** 2025-03-31

**Authors:** Yongjie Mei, Jianguo Chen, Yingyu Chen, Changmin Hu, Xi Chen, Aizhen Guo

**Affiliations:** 1 National Key Laboratory of Agricultural Microbiology, Hubei Hongshan Laboratory, College of Veterinary Medicine, Huazhong Agricultural University, Wuhan 430070, China; meiyongjie789@163.com (Y.M.); chenjg@mail.hzau.edu.cn (J.C.); chenyingyu@mail.hzau.edu.cn (Y.C.); chm@mail.hzau.edu.cn (C.H.); chenxi@mail.hzau.edu.cn (X.C.); 2 The Cooperative Innovation Centre for Sustainable Pig Production, Hubei International Scientific and Technological Cooperation Base of Veterinary Epidemiology, Wuhan 430070, China; 3 Ministry of Agriculture and Rural Affair, Key Laboratory of Development of Veterinary Diagnostic Products, Wuhan 430070, China

**Keywords:** porcine reproductive and respiratory syndrome virus, NADC34-like virus, pathogenicity, Nsp2, GP5

## Abstract

Porcine Reproductive and Respiratory Syndrome Virus (PRRSV) is one of the most significant infectious agents threatening the global pig industry. Due to its high mutation and recombination rates, the prevalence of PRRSV in domestic pig populations is complex. To better understand the epidemiology of PRRSV, we conducted a large-scale investigation in eastern China, focusing on pig farms with a history of high abortion rates. A total of 14,934 pig samples were collected from 11 sow farms and 53 fattening farms across three provinces. Among these, 13.0% of the collected samples tested positive for PRRSV, with specific prevalence rates of 19.7% in sows and 12.4% in piglets. Genetic evolution analysis of the GP5 gene from 43 PRRSV strains identified in this study revealed that NADC30-like, NADC34-like, and HP-PRRSV were the predominant lineages in domestic pig farms. The NADC30-like genotype was the most dominant and had evolved into three subgenotypes, while the NADC34-like strains had diverged into two subgenotypes. Further analysis of the Nsp2 gene from 18 strains indicated that the NSP2 gene of multiple NADC34-like strains was closely related to that of the NADC30-like, suggesting that the NADC34-like strains are primarily recombinant viruses. Sequence comparison of the Nsp2 gene showed that both NADC30-like and NADC34-like viruses share 111 amino acid deletions at positions 322–433 and 21 amino acid deletions at positions 539–558 in the Nsp2 gene coding region. For the first time, the pathogenicity of a representative NADC34-like virus isolated in China was evaluated in pregnant sow. The results showed that infected sows exhibited an increased body temperature, ear cyanosis, and typical edema and cyanosis of the external genitalia. Moreover, all infected sows experienced miscarriage, with 100% of the aborted piglets being stillbirths exhibiting a high virus load. These findings indicate that this NADC34-like virus is highly virulent to sows.

## 1. Introduction

Porcine Reproductive and Respiratory Syndrome Virus (PRRSV) was first identified in the Netherlands in 1991 and the United States in 1992. The initially isolated strains were named Lelystad Virus and Swine Inflammation and Respiratory Syndrome Virus (ATCC VR-2332) [1]. Currently, PRRSV is primarily classified into two genotypes: PRRSV-1 and PRRSV-2 [2]. Due to the high genetic variability of PRRSV, a classification system based on the complete sequence of the ORF5 gene has been established [3]. According to this system, PRRSV-1 is divided into three subgenotypes (subtypes 1, 2, and 3), while PRRSV-2 is classified into nine subgenotypes, each of which is further divided into several lineages [4].

PRRS was first reported in China in 1995, and the CH-1a strain was isolated from aborted fetuses in 1996. The CH-1a strain, closely related to the North American isolate, belongs to PRRSV-2. Epidemiological investigations have shown that the CH-1a strain was predominantly prevalent in China from 1995 to 2005 [5]. In 2006, an outbreak in the Chinese pig population was identified as being caused by a new highly pathogenic PRRSV strain (HP-PRRSV), which caused devastating damage to the pig industry with a fatality of 20% [6]. HP-PRRSV is characterized by a unique 30 amino acid deletion in the Nsp2 gene coding region [7]. Since 2006, HP-PRRSV strains have become one of the prevalent genotypes in China [8].

The more recent NADC30-like PRRSV was first identified in China in 2013 [9]. Currently, the NADC30-like strains are widely prevalent in China [10]. The Nsp2 gene of the NADC30-like PRRSV contains 131 amino acid deletions [11]. In addition, compared with other PRRSV genotypes, the NADC30-like PRRSV exhibits more recombinant genotypes resulting from recombinations with various types of strains, including HP-PRRSV strains, vaccine strains (JXA1-P80), classical PRRSV (VR2332) [11,12]. These strains display diverse pathogenic characteristics, with some exhibiting high pathogenicity comparable to HP-PRRSV [9,13], while others show moderate pathogenicity similar to the NADC30 prototype virus [9]. This variability in pathogenicity may primarily result from extensive mutations and recombinations [14].

Furthermore, the NADC34-like PRRSV has recently emerged as a significant threat to the pig farming industry in the United States. Most NADC34-like viruses, which first appeared in the United States in 2014, are highly pathogenic in sow herds and cause abortion ‘storms’ in sow. This NADC34-like PRRSV was first reported in Northeast China in 2017, and its isolation rate has gradually increased since then [15]. Evolutionary analysis revealed that the Nsp2 protein has the same 100-amino-acid deletion as IA/2014/NADC34, a strain identified in the United States in 2014 [10]. The pathogenicity of these NADC34-like strains isolated in China has been evaluated in piglets, showing significant differences in pathogenicity among the strains [16]. However, the pathogenicity of NADC34-like strains circulating in China in pregnant sows has not been fully evaluated.

To better understand PRRSV prevalence in China, we conducted a survey on pig farms with a history of high abortion rates from Jiangsu, Zhejiang, and Anhui Provinces, using routine surveillance samples from sows, piglets, and boars during 2022 and 2023. In addition, we evaluated the pathogenicity of a representative NADC34-like virus isolate in pregnant sows. The results provided valuable insights for PRRSV control in China.

## 2. Materials and Methods

### 2.1. Sample Collection

To understand the prevalence of PRRSV in pig herds from large-scale breeding enterprises in China with a history of abortion rates exceeding 2%, routine surveillance samples of pigs were received by this laboratory from 11 sow farms (with a stock size of 72,000 heads) and 53 fattening farms (with a stock size of 640,000 heads) located in Jiangsu, Zhejiang, and Anhui Provinces during 2022–2023. The samples included aborted sow blood, aborted fetal tissues, operating fluid from parturient piglets, blood samples from weaning weak piglets, blood samples from nursing piglets, lymph nodes from deceased piglets, testicular fluid, and semen samples. A total of 14,934 samples were collected (Table 1) for PRRSV detection using TaqMan real-time RT-PCR, as described previously. Primers and probes were selected and designed from conserved regions of the ORF7 gene. ORF7-F: GGGGAATGGCCAGYCAGTCAA; ORF7-R: GCCAGRGGAAAATG KGGCTTCTC; and probe: FAM-CTGGGYARGATYATCGCCCAGCA-BHQ1. Meanwhile, portions of the PRRSV-positive samples were used for virus isolation and GP5 and Nsp2 gene amplification for genetic evolution analysis.

### 2.2. Cell Culture and Virus Titration

The MARC-145 cell lines were stored in the laboratory. The MARC-145 cells were cultured in RPMI 1640 medium (Gibco, Santa Clara, CA, USA) supplemented with 10% fetal bovine serum (FBS) (Gibco, USA), 1% penicillin–streptomycin (Invitrogen, Carlsbad, CA, USA), 10 mM HEPES, and 10 mM essential amino acids (HyClone, Logan, UT, USA). The Marc-145 cells were cultured in Dulbecco’s modified Eagle’s medium (DMEM) (Gibco, USA) containing 10% FBS and 1% penicillin–streptomycin. All cells were cultured in a 37 °C incubator with 5% CO_2_. The peripheral blood mononuclear cells (PBMCs) were isolated from the PRRSV-negative piglet by density centrifugation. As described previously, the isolated PBMCs were cultured in Dulbecco’s modified Eagle’s medium (DMEM) (Gibco, USA) containing 10% FBS and 1% penicillin–streptomycin [17].

Briefly, the tissues were homogenized in PBS using a tissue grinder (Tissuelyser-FEIII, Jingxin, Shanghai, China). The tissue homogenate was spun at 4860 g for 10 min. Then, 1 mL of the supernatant was collected and inoculated into the MARC-145 cells in a T25 flask, which was then replaced with 5 mL of fresh medium after one hour of adsorption. Then, the supernatant and cells were collected upon the occurrence of cytopathic effect (CPE) including pyknosis and detachment. The isolated virus will be further verified by RT-PCR for PRRSV. To determine the titers of the virus isolate, the amplified NADC34-like virus was serially diluted 10-fold and inoculated into PBMC cells cultured in a 96-well cell culture plate. Negative control groups with PBS were included in the titration procedure. After 5 days of incubation, when the CPE developed, the number of wells with CPE was recorded, and the virus titers (TCID50) of PRRSV isolates were calculated based on three independent replicates using the Reed–Muench formula.

### 2.3. RT-PCR

To isolate viral RNA, 0.2 mL samples containing virus were used for RNA extraction according to the MagaBio plus virus DNA/RNA purification kit III (Hangzhou Boji Biotechnology Co., Ltd., Hangzhou, China). a total volume of 30 ul isolated RNA was subjected to reverse transcription by using RNA reverse transcription Kits (Jinweizhi Co., Ltd., Suzhou, China). In order to analyze the prevalence and genetic evolution of PRRSV, the primers were designed for the GP5 and NSP2 genes of PRRSV as described in the previously published literature: GP5-F: ATGTTGGGGAGATGCTTGACCGC; GP5-R: CTATGGACGACCCCATTGTTCCG; NSP2-F: AAGAGAGCTAGAAGAGCACGTG; and NSP2-R: GATGCCAAGCCTAAGCAGAAAC [18]. Both the GP5 and Nsp2 genes were amplified by RT-PCR. Then, the RT-PCR products were purified, commercially sequenced at Tsingke Biotech Co., Ltd. (Shanghai, China), and analyzed.

### 2.4. Phylogenetic Analysis

Phylogenetic analysis of PRRSV isolates, based on the GP5 and Nsp2 gene sequences obtained in this study and the reference strains retrieved from GenBank, was conducted using the neighbor-joining (NJ) and maximum likelihood NJML methods contained in MEGA6 software version 10.25. The NJ method was applied for 1000 repeats, while the ML method was applied for 100 repeats. Meanwhile, the amino acid sequences of GP5 and Nsp2 were compared using the MEGALIGN function in DNAStar software version 7.1.

### 2.5. Animal Experiment

To evaluate the pathogenicity of the NADC34-like PRRSV isolates in sows, one representative NADC34-like virus strain (NAD2022) isolated from the blood of an aborted sow was chosen to infect the pregnant sows. This experiment was reviewed, approved, and monitored by the Huazhong Agricultural University Science Ethics Committee (Protocol Permit No. HZAUSW-2025-0004).

In detail, six PRRSV sero-negative pregnant sows were divided into two groups, with three sows in each group. The infected group of pigs (gestational age of 3 months) was inoculated intramuscularly with a dose of 10^6^ TCID50 diluted in 1 mL of PBS containing NAD2022, while the control group received an intramuscular injection of PBS. All the infected pigs and control pigs were kept in separate rooms with the same controlled housing conditions, including temperature (20–22 °C), feeding, and biosecurity level. After challenging, the rectal temperature of the pigs was recorded at about 9:00 AM every day, and their clinical signs, such as appetite, mental status, and respiratory status, were observed. About 2 mL of blood samples were collected in a 15 mL tube at every two days post-infection for serum isolation. The collected blood samples were kept in a 4 °C refrigerator for 4 h, and then the serum was collected after centrifugation at 1000× *g*. The virus load in serum was detected by real-time RT-PCR, as described in Section 2.2. The aborted piglets were necropsied, and different tissue samples from hearts, spleens, livers, lungs, lymph nodes, kidneys, umbilical cords, and placentas were collected to detect the virus loads.

### 2.6. Statistical Analysis

All data in this study are presented as mean ± standard deviation (SD) and were analyzed using a one-way analysis of variance (ANOVA) in GraphPad Prism version 6.0 (GraphPad Software, Inc., San Diego, CA, USA). Statistical significance was set at *p* < 0.05, and highly significant differences were defined as *p* < 0.01. The accurate confidence intervals for the positive rate were calculated as described previously [19].

## 3. Results

### 3.1. Prevalence of PRRSV in Investigated Eastern China

The overall prevalence was 13.0% (95% CI: 12.5%, 13.6%) among the 14,934 surveillance samples. Significant differences in prevalence were observed among samples from sows, piglets, and boars (*p* < 0.05). Aborted sows showed the highest prevalence at 19.7% (95% CI: 18.4%, 20.9%), followed by the associated piglet samples with a prevalence of 12.4% (95% CI: 11.7%, 13.1%), suggesting potential transmission from sows to their piglets. Boar semen samples had the lowest prevalence of 0.5% (95% CI: 0.2%, 1.0%) (Table 2). Furthermore, sample types significantly affected the prevalence rates. Among the aborted sows, blood samples exhibited the highest prevalence, which was significantly higher than that of the swabs (*p* < 0.05). However, in the piglets, tissue samples displayed the highest prevalence rates, significantly higher than the other two sample types, blood and testicular fluids (*p* < 0.05).

In addition, the prevalence rates were compared among the provinces (Table 3). The overall prevalence rate of PRRSV in Jiangsu (13.7%) is higher than Anhui (11.9%) and Zhejiang (12.0%) Provinces. Furthermore, the prevalence rate of the sows was the highest prevalence rates at 22.3% (Jiangsu), 18.5% (Anhui), and 15.0% (Zhejiang), followed by that of the piglets at 13.5% (Jiangsu), 10.0% (Anhui), and 11.6% (Zhejiang), respectively, while the boar semen samples had the least prevalence of 0.7% (Jiangsu), 0% (Anhui), and 0% (Zhejiang).

### 3.2. Virus Isolation and Identification

To isolate the PRRSV virus, the PRRSV-positive tissue samples from the aborted sows were homogenized and spun at 4860× *g* for 10 min, and the supernatant was inoculated into the MARC-145 cells for virus isolation. A total of four PRRSV strains were successfully isolated and designated as PRRSV-LP, PRRSV-DYS, PRRSV-DY1A, and PRRSV-NAD2022. To characterize these viruses, the GP5 gene was amplified and sequenced. The phylogenetic analysis of GP5 showed that the PRRSV-LP belongs to the NADC30-like genotype, while the PRRSV-DYS, PRRSV-DY1A, and PRRSV-NAD2022 show a close relationship with the NADC34-like viruses. Notably, all three NADC34-like viruses were isolated from the aborted sows. Analysis of the Nsp2 gene showed that these three viruses are closely related to the NADC30-like virus, which suggested the three NADC34-like viruses are reassortant viruses. To further investigate the pathogenicity of NADC34-like viruses isolated in China, PRRSV-NAD2022, which exhibits distinct genetic differences from the US NADC34 virus, was selected for further animal studies.

### 3.3. Evolutionary Analysis of GP5 Gene of PRRSV

**Figure 1:**
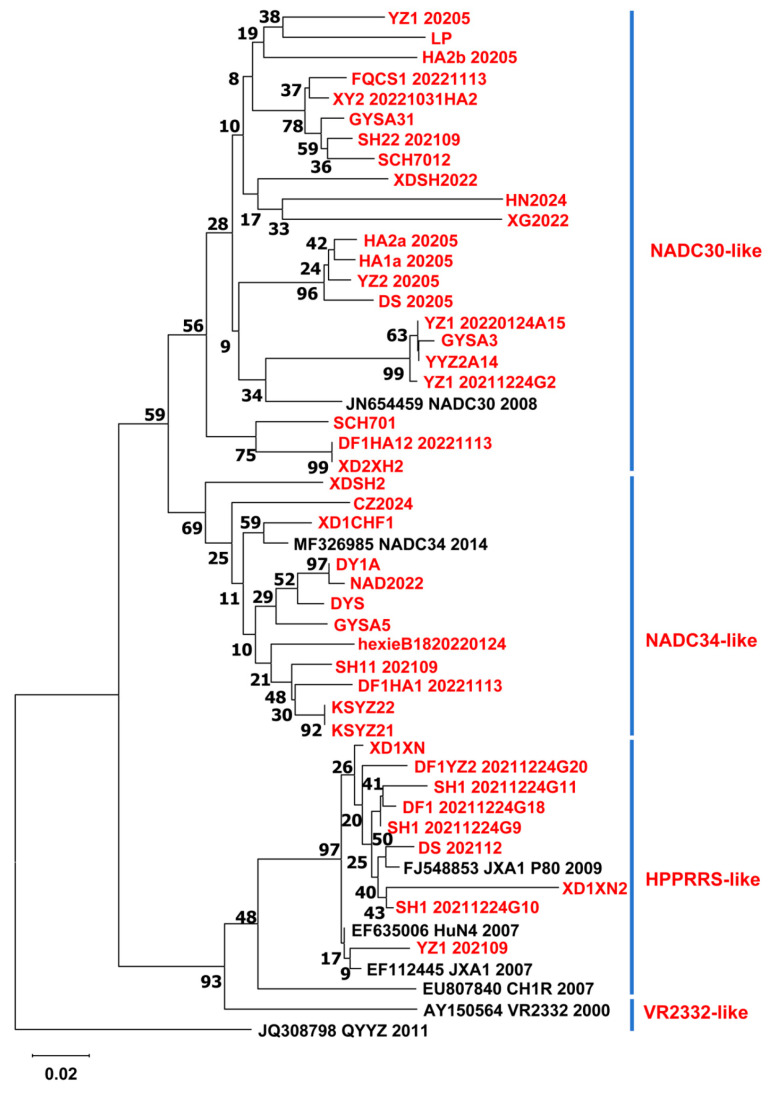
Genetic evolution analysis of the PRRSV GP5 gene. Phylogenetic trees were generated using the neighbor-joining method in MEGA6 software version 10.25. The viruses identified in this study are highlighted in red, and the PRRSV reference strains are highlighted in black. The scale bar represents the evolutionary distance between virus pairs.

To perform genetic evolution analysis, the ORF5 gene of PRRSV was amplified and sequenced from 43 positive samples. Phylogenetic analysis showed that 22 PRRSV strains were NADC30-like viruses, while 12 PRRSV strains belonged to NADC34-like viruses. The remaining nine strains clustered with HP-PRRSV (Figure 1). Furthermore, the NADC30-like virus isolates were grouped into three lineages: one closely related to the original NADC30 virus and two that were more distantly related to it. Similarly, NADC34-like viruses identified in this study have evolved into two lineages (Figure 1). Five strains clustered within the same branch as the American NADC34 reference strain, but the remaining five viruses exhibited a certain genetic distance from it (Figure 1). The other nine PRRSV strains belonged to the HP-PRRSV branch (Figure 1).

### 3.4. Comparative Analysis of GP5 Amino Acid Sequences of Local PRRSV Strains

**Figure 2:**
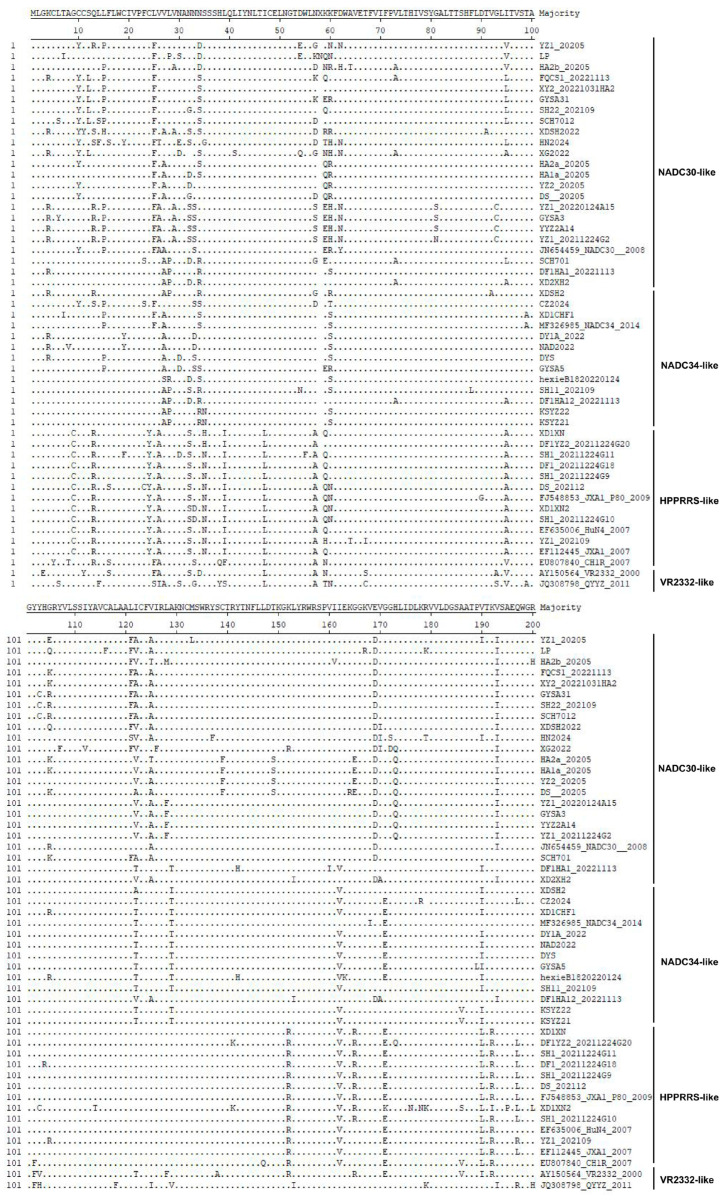
Comparison of GP5 amino acid sequences of 43 PRRSV strains. The GP5 protein sequences of PRRSV were aligned using Lasergene 7.0. The dot indicates the residues that match the consensus sequences.

Comparative analysis of the GP5 amino acid sequences of the 43 local PRRSV strains with reference strains showed that the homology of GP5 amino acid sequences among different branches ranged from 79% to 99%, with characteristic mutations in multiple regions of this gene. The GP5 protein is a membrane protein with a signal peptide and transmembrane, extracellular, and intracellular regions. The extracellular region of GP5 is one of the areas with many amino acid mutations, among which the glycosylation modifications at positions 44–46 NLT and 51–53 NGT are the most conserved among all PRRSV strains [20]. However, in 14 NADC34-like and NADC30-like strains, GP5 has an additional NXS glycosylation modification site at positions 32–34, mainly NNS or NSS. Most HP-PRRSV viruses have an NNS glycosylation modification site at positions 33–35 of GP5. The 39th position of GP5 in NADC34-like and NADC30-like viruses is L39, while in HP-PRRSV, it is I39. The 47th position of GP5 in NADC34-like and NADC30-like viruses is I47, while in HP-PRRSV, it is L47 (Figure 2). The most variable region within the extracellular domain is located at positions 57–60. The 57th position of GP5 in NADC34-like viruses is N, whereas in NADC30-like viruses, it is mostly D/K/S amino acids, which is not conservative. In contrast, HP-PRRSV has A at this position, which is relatively conservative. The 59th and 60th amino acids of GP5 in the NADC34-like virus are KS, while HP-PRRSV has QK, which may be related to the antigenic differences among different branch viruses (Figure 2).

There are also some amino acid mutations in the intracellular region of the GP5 protein (Figure 2). For instance, the 151st amino acid of NADC34-like and NADC30-like strains is K, while that in HP-PRRSV is R. The 161st amino acid of the NADC30-like strain GP5 is I, while that in other PRRS viruses is mostly V. The 164th position of the NADC34-like and NADC30-like strains is G, while that in other PRRS viruses is mostly R. The 168th position of the NADC30-like strain is mostly D, while that of the NADC34-like and other strains is E. The 170th position of the NADC30-like strain is G, while that in the NADC34-like and other strains is E. The 189th positions of the NADC34-like and NADC30-like strains are mostly V or I, while in HP-PRRSV, it is mostly L. The 192nd position of the NADC30-like strain is mostly I, while that in the NADC34-like and other strains is V. The 196th and 200th positions of the NADC30-like and NADC34-like strains are mostly Q/P, while those in HP-PRRSV are mostly L (Figure 2).

### 3.5. Evolutionary Analysis of Nsp2 Gene of Local PRRSVstrains

**Figure 3:**
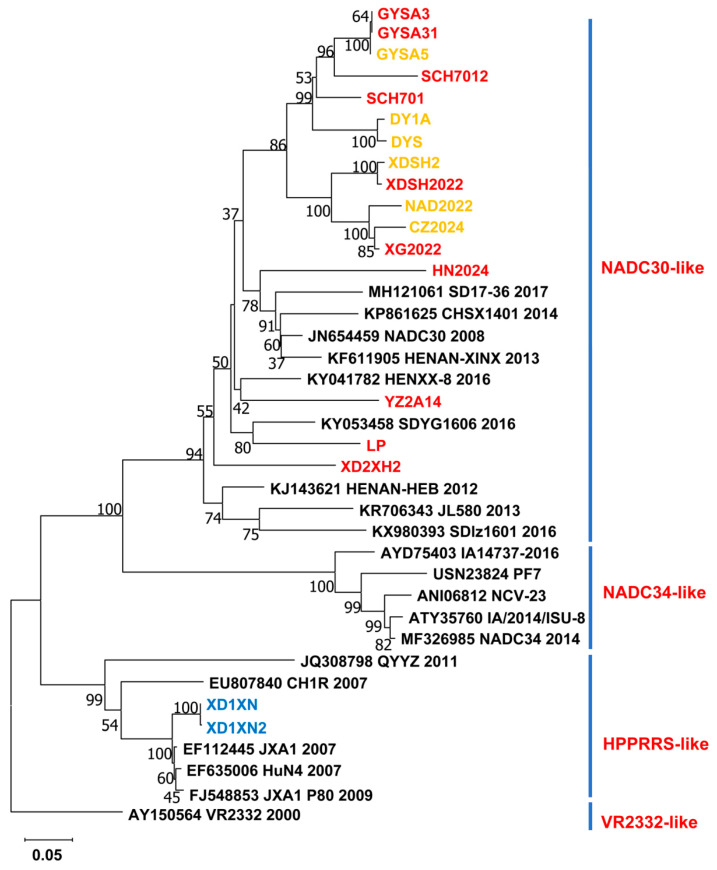
Genetic evolution analysis of the NSP2 gene of 18 PRRSV strains. Phylogenetic trees were generated using the neighbor-joining method in MEGA6 software version 10.25. The viruses identified in this study are highlighted in color: the NADC30-like strains (based on the GP5 phylogenetic tree) in red, the NADC34-like strains in yellow, the HP-PRRSV strains in blue, and the PRRSV reference strains in black. The scale bar represents the evolutionary distance between virus pairs.

The Nsp2 gene is one of the most variable genes in the PRRSV genome. Among 43 strains identified in this study, the full-length Nsp2 gene was successfully amplified and sequenced from only 18 samples. The nucleotide sequence homology of the Nsp2 gene among these 18 strains ranges from 60.4% to 99.9%. In the phylogenetic tree of Nsp2 sequences from these 18 strains, most of the strains were closely related to the NADC30-like strain, and only two strains (XD1XN and XD1XN2) had a close relationship with the HP-PRRSV-like strain (Figure 3). For the strains GYSA5, DY1A, DYS, XDSH2, NAD2022, and CZ2024, the GP5 gene belonged to the NADC34-like genotype, while the Nsp2 gene showed a close relationship to the NADC30-like genotype. This suggests that the currently prevalent NADC34-like PRRSVs in China are mainly recombinant strains, with the gene backbone of most NADC34-like strains likely dominated by NADC30-like elements.

### 3.6. Comparative Analysis of NSP2 Amino Acid Sequences in the 18 PRRSV Strains

**Figure 4:**
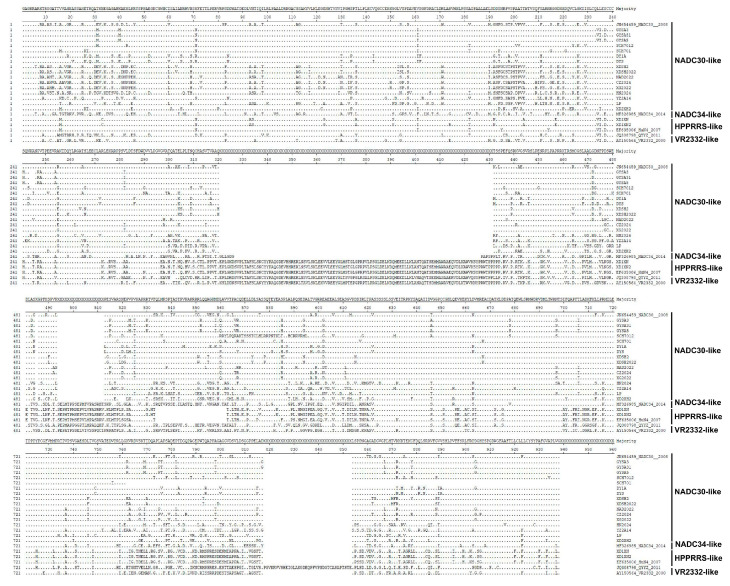
Comparison of Nsp2 amino acid sequences of PRRSV strains. The Nsp2 protein sequences of PRRSV strains were aligned using Lasergene 7.0. The dots indicate the residues that match the consensus sequences, while the blanks indicate the deletion regions.

The amino acid similarity of the NSP2 protein of the 18 PRRSV strains ranges from 52.7% to 99.8%. The comparison results showed that most NSP2 proteins belong to the NADC30-like branch with similar deletion patterns, which are 111 amino acid deletions between amino acid positions 322 and 433 and 21 amino acid deletions between amino acid positions 539 and 558 (Figure 4). However, the HP-PRRSV-like viruses XD1XN1 and XD1XN2 are similar to HP-PRRSV, with only 29 amino acid deletions between amino acid positions 539 and 568 (Figure 4). Therefore, based on the evolutionary analysis and amino acid deletion patterns of the NSP2 gene coding region sequences, the PRRSV strains circulating in China are predominantly NADC30-like.

### 3.7. Pathogenicity of the NADC34-like PRRSV in Pregnant Sows

**Figure 5:**
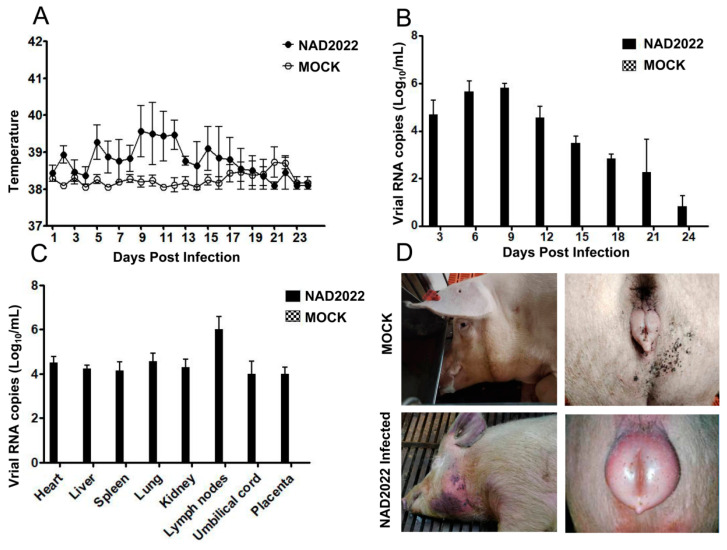
Pathogenicity of the NADC34-like PRRSV (NAD2022) in pregnant sows: (**A**) changes in body temperature of the infected sows and mock group; (**B**) viral load in serum of the infected sows and mock group; (**C**) virus distribution in the stillborn piglets; and (**D**) clinical signs in the infected sows and mock group.

The pathogenicity of NADC34-like PRRSV isolated in China has been evaluated in piglets; however, its pathogenicity in pregnant sows remains largely unclear. To assess the pathogenicity of NADC34-like PRRSV, a representative NADC34-like isolate (PRRSV-NAD2022) was used to infect pregnant sows. The infected sows exhibited a significant increase in body temperature, reaching 39 °C on the third day after infection, and maintaining between 39 and 40 °C until 14 days after infection (Figure 5A). In contrast, the body temperature of the pigs in the control group remained within normal ranges throughout the experiment.

The infected sows showed typical symptoms of PRRSV infection, including cyanosis of the ears, depression, coarse and messy fur, and typical subcutaneous hemorrhagic cyanosis on the neck and ventral skin. Three infected sows exhibited typical edema and cyanosis of the external genitalia, characterized by a purple-black color and inflammatory secretions. In contrast, no significant clinical signs were observed in the control group of sows (Figure 5D).

All three sows infected with the NADC34-like virus experienced miscarriages, with two sows delivered 9 and 11 stillborn piglets, respectively, 6 to 7 days earlier than expected. The third infected sow expelled 13 stillborn piglets at 8 days after the due date of delivery. To determine the distribution of NADC34-like virus in different organs of stillborn piglets, viral RNA was extracted from the hearts, spleens, livers, lungs, lymph nodes, kidneys, umbilical cords, and placentas, and the virus load was detected using qPCR. As a result, the NADC34-like virus was detected in different organs of stillborn piglets, with the lymph nodes having the highest viral load (Figure 5C). All piglets born to the sows in the control group survived without any stillbirths. These results indicated that the NADC34-like virus caused 100% miscarriage and 100% stillbirth among the infected pregnant sows.

To investigate viremia in sows following NADC34-like virus infection, serum samples were collected at various time points after infection. The results showed that the NADC34-like virus was present at high levels in the early stages of infection in the pregnant sows, and the virus levels gradually decreased over time (Figure 5B). In one month after infection, the virus level in the serum had significantly declined.

## 4. Discussion

PRRSV is one of the most important viruses infecting pigs, which causes significant economic losses to the global pig industry [10]. As an RNA virus, the PRRSV frequently undergoes mutations and recombinations among different genotypes, which causes the complex prevalence of PRRSV in pig herds [21]. In this study, surveillance findings of PRRSV in pig herds of large-scale breeding enterprises with a high level of abortion in eastern China provide valuable insights for understanding the transmission dynamics of PRRSV and implementing early intervention measures at critical control points.

Although the PRRSV is able to infect pigs of all ages, the infection mainly causes severe reproductive failure in pregnant sows. Moreover, the duration of PRRSV infection can last for 3 to 4 months in pigs [22]. PRRSV is able to cross the placenta and infect fetuses, causing abortion and weak-born piglets [23]. In this study, the prevalence of PRRSV infection was 19.7% in the sows and 17.5% in the associated piglets, which is higher than that of the boar samples, suggesting that PRRSV is a significant threat to pregnant sows and their associated piglets in the investigated pig herds. PRRSV is primarily transmitted in pigs through direct contact or exposure to contaminated semen, secretions, and blood [24].

Therefore, we checked the prevalence of PRRSV in boar semen and testicular fluids of male piglets. In this study, we found that the prevalence of PRRSV in boar semen is relatively low at 0.5%, and most boars did not show clinical signs of PRRSV infection. Given that the clinical manifestations of PRRSV infection in boars are often subtle [25], this low prevalence can easily be overlooked, potentially facilitating PRRSV transmission through semen and resulting in more severe economic losses. Phylogenetic analysis of the GP5 gene showed that the PRRSV detected in boars belongs to NADC30-like viruses, suggesting potential transmission through contaminated vectors such as equipment, instruments, or personnel. Therefore, routine PRRSV surveillance in boar semen is crucial for effective PRRSV control in sow populations.

It has previously been confirmed that PRRSV can be transmitted from the infected sows either vertically through the placenta or horizontally from the sows via contaminated air. In accordance with these findings, this study demonstrated that the testicular fluids of castrated male piglets aged 3–5 days had a prevalence of 11.4%. Therefore, the farms can implement early, convenient, and cost-effective interventions for PRRSV infection control by monitoring the testicular fluids of castrated piglets.

Phylogenetic analysis of the GP5 gene from 43 PRRSV strains revealed that NADC30-like, NADC34-like, and HP-PRRSV strains are prevalent in domestic pig farms. Among these, the NADC30-like strain is currently the dominant strain and has evolved into three distinct sub-branches, suggesting rapid mutation within the domestic pig population. Similarly, the NADC34-like strain has diverged into two branches: one clusters with the American NADC34 reference strain, while the other exhibits significant genetic divergence from NADC34, indicating the ongoing evolution of NADC34-like viruses in Chinese pig populations (Figure 1). Furthermore, evolutionary analysis of the NSP2 gene from 18 PRRSV strains demonstrated that the NSP2 gene of NADC34-like strains is closely related to that of NADC30-like viruses. Both NADC30-like and NADC34-like viruses share 111 amino acid deletions at positions 322–433 and 21 amino acid deletions at positions 539–558 in their NSP2 sequences. This suggests that NADC34-like strains are recombinant viruses and that frequent reassortment among different PRRSV lineages occurs in pig herds.

Before 2020, the main prevalent strains of PRRSV in China could be divided into four lineages: 8.7, 5.1, 3.5, and 1.8 (NADC30-like) [10,26]. The NADC34 strain, first identified in the United States in 2014, caused a widespread miscarriage storm in sows [27]. The PRRSV isolate was designated IA/2014/NADC34 [15]. Subsequently, between 2015 and 2017, NADC34 spread to Peruvian pig herds [28]. In 2017, NADC34-like strains emerged in pig herds in South Korea, where the virus was identified as a recombinant strain resulting from genetic recombination between NADC34 and NADC30 [29]. In the same year, NADC34-like strains were detected in China, and further sequence analysis confirmed that they were also recombinant viruses [30]. Currently, NADC34-like strains have become one of the predominant strains in China. The NADC34-like PRRSV, which initially emerged in the United States, was a highly pathogenic strain that caused severe miscarriage storms in pregnant sows and high mortality in piglets [31]. Subsequent PRRSV outbreaks involving NADC34-like strains have shown significant differences in pathogenicity, with some strains not causing typical PRRSV symptoms such as fever, indicating that the pathogenicity of the NADC34-like virus varies significantly, likely due to recombination and other factors.

Since the emergence of the NADC34-like PRRSV in China, various recombinant NADC34-like viruses have arisen through recombination with other PRRSV strains, indicating that the NADC34-like viruses isolated in China exhibit significant variations in pathogenicity due to the complexity of the recombination events. However, the pathogenicity of the NADC34-like virus circulating in China, particularly in pregnant sows, has not been thoroughly investigated. In this study, we first evaluated the pathogenicity of the NADC34-like PRRSV strain from China in pregnant sows. The results showed that all infected sows experienced abortion and 100% stillbirths, indicating that NADC34-like viruses are highly pathogenic to sows. Similar to the American isolate, the NADC34-like PRRSV prevalent in China poses a significant threat to pregnant sows, causing abortions and resulting in substantial economic losses.

## 5. Conclusions

In conclusion, this study revealed varying levels of PRRSV prevalence in large-scale pig farms with a history of sow abortion. The use of testicular fluids from castrated piglets for PRRSV surveillance has proven to be an early, convenient, and cost-effective method for infection control. Furthermore, three PRRSV genotypes—NADC30-like, NADC34-like, and HP-PRRSV—were identified as prevalent in domestic pig farms. For the first time, the pathogenicity of a novel NADC34-like virus isolated in China was evaluated in pregnant sows, demonstrating that this virus is highly pathogenic to them. These findings highlight the urgent need for a commercial NADC34-like vaccine to enhance PRRSV control strategies in China.

## Figures and Tables

**Table 1:** The sample information.

Animals	Sample Kinds	Sample No			Total
		Jiangsu	Anhui	Zhejiang	
Sows	Blood	816	414	380	1610
	Swab	1100	560	440	2100
	Tissue	126	117	69	312
Piglets	Blood	486	198	92	776
	Tissue	324	216	111	651
	Testicular fluid	4820	1809	1207	7836
Boars	Semen	1218	264	167	1649
Total		8890	3578	2466	14,934

**Table 2:** PRRSV prevalence of the samples from sows, piglets, and boars. The confidence interval (CI) for the prevalence rate was calculated as previously described [19], where “Average” represents the positive rate, while “Lower” and “Upper” denote the confidence interval boundaries.

Pig Types	Sample Types	Sample No	Positive No	Prevalence % (95% CI: Down, Upper)		
				Average	Down	Upper
Sows	Blood	1610	392	24.4%	21.6%	27.2%
	Tissue	312	61	19.6%	15.3%	24.4%
	Swab	2100	338	16.1%	14.5%	17.7%
	Subtotal	4022	791	19.7%	18.4%	20.9%
Piglets	Blood	776	102	13.1%	10.8%	15.7%
	Tissue	651	149	22.9%	19.7%	26.3%
	Testicular fluid	7836	895	11.4%	10.7%	12.1%
	Subtotal	9263	1146	12.4%	11.7%	13.1%
Boars	Semen	1649	8	0.5%	0.2%	1.0%
Total		14,934	1945	13.0%	12.5%	13.6%

**Table 3:** Comparison of prevalence among three provinces. “Pos No” represents the positive number, while “Pre%“ denotes the prevalence rate.

Animals	Sample Kinds	Prevalence in Three Provinces								
		Jiangsu			Anhui			Zhejiang		
		Test No	Pos No	Pre%	Test No	Pos No	Pre%	Test No	Pos No	Pre%
Sows	Blood	816	230	28.2	414	98	26.8	380	64	16.8
	Tissue	126	30	23.8	117	19	16.2	69	12	17.4
	Swab	1100	196	17.8	560	85	15.2	440	57	13.0
	Subtotal	2042	456	22.3	1091	202	18.5	889	133	15.0
Piglets	Blood	486	65	13.4	198	26	13.1	92	11	12.0
	Tissue	324	85	26.2	216	44	20.4	111	20	18.0
	Testicular fluid	4820	610	12.7	1809	153	8.5	1207	132	10.9
	Subtotal	5630	760	13.5	2223	223	10.0	1410	163	11.6
Boars	Semen	1218	8	0.7	264	0	0	167	0	0
	Total	8890	1224	13.7	3578	425	11.9	2466	296	12.0

## Data Availability

The original contributions presented in this study are included in the article. Further inquiries can be directed to the corresponding author.
